# Analytical Characterization of the Widely Consumed Commercialized Fermented Beverages from Russia (Kefir and Ryazhenka) and South Africa (Amasi and Mahewu): Potential Functional Properties and Profiles of Volatile Organic Compounds

**DOI:** 10.3390/foods10123082

**Published:** 2021-12-11

**Authors:** Konstantin V. Moiseenko, Olga A. Glazunova, Olga S. Savinova, Betty O. Ajibade, Oluwatosin A. Ijabadeniyi, Tatyana V. Fedorova

**Affiliations:** 1 A.N. Bach Institute of Biochemistry, Research Center of Biotechnology, Russian Academy of Sciences, 119071 Moscow, Russia; mr.moiseenko@gmail.com (K.V.M.); olga.a.glas@gmail.com (O.A.G.); savinova_os@rambler.ru (O.S.S.); 2 Department of Biotechnology and Food Technology, Faculty of Applied Sciences, The Durban University of Technology, Durban 4001, KwaZulu-Natal Province, South Africa; bettyajibade@gmail.com (B.O.A.); oluwatosini@dut.ac.za (O.A.I.)

**Keywords:** kefir, ryazhenka, amasi, mahewu, antioxidant properties, ACE inhibition, fatty acid profile, volatile organic compounds, GC-MS

## Abstract

In this study, four commercialized indigenous fermented beverages most highly consumed in Russia (kefir and ryazhenka) and South Africa (amasi and mahewu) were analyzed for their potential health-promoting properties and flavor-forming volatile organic compounds (VOC). The analysis of antioxidant capacity demonstrated superiority of dairy-based beverages (kefir, ryazhenka and amasi) over the corn-based mahewu; however, mahewu outperformed dairy-based beverages in terms of its potential antihypertensive effect (i.e., the ability to inhibit angiotensin I converting enzyme). The fatty acid (FA) content of kefir and ryazhenka were more diverse compared to that of amasi, but included a lesser amount of branched chain FA. In terms of calculated FA nutritional indices (e.g., indices of atherogenicity and thrombogenicity), kefir and ryazhenka performed similarly and significantly better than amasi. The agreement between beverages theoretical flavor profiles, which was obtained based on the flavors of individual VOC, and consumers’ flavor perception allow hypothesizing about the contribution of detected VOC to the overall products’ flavor. The obtained data expand current knowledge regarding traditional fermented beverages and their values in terms of national dietary recommendations. Additionally, reported VOC profiles will promote the inclusion of traditional fermented beverages into the rations based on the flavor pairing concept (which is controversial but widely applied).

## 1. Introduction

The origin of food fermentation, the production of foodstuff through the microbially mediated biochemical modification of edible and sometimes inedible raw material, dates at least 6000 years into the past [1]. Currently, many traditional fermentation processes have been industrialized, and a wide spectrum of indigenous or traditional fermented foods can easily be found both on the local and global market [2]. Although nowadays food fermentation has lost its paramount importance as a preservation method, fermented food is still widely consumed for its high nutritional value, pleasing sensory attributes and potential health-promoting properties [3].

Both in Russia and South Africa, fermented dairy products traditionally form a substantial part of the human diet. Besides internationally distributed products, such as yoghurt, both these countries have a number of purely national foods: kefir and ryazhenka in Russia [4], and amasi in South Africa [5]. In addition, in South Africa, a maize-based fermented drink, mahewu, is extremely popular, especially in rural areas [6].

In short, kefir is a traditional fermented milk originating from the Caucasus Mountains. However, nowadays it is one of the most popular dairy beverages in Russia. Both commercial and traditional preparation technology implies the use of a starter obtained with “kefir grains”, a complex consortium of microorganisms including *Lactococcus* spp., *Lactobacillus* spp., *Leuconostoc* spp., yeasts and acetic acid bacteria [4]. Ryazhenka is another Russian traditional dairy beverage, commercialized and wildly distributed on the national market. The main feature of ryazhenka is the use of baked milk, while the starter used is a traditional yogurt’s starter containing *Lactobacillus bulgaricus* and *Streptococcus thermophilus* [4]. Amasi is indigenous fermented milk produced in South Africa on an industrial scale. Commercial amasi is made from cow’s milk using mesophilic cultures containing *Lactococcus lactis* subsp. lactis, *Lactococcus lactis* subsp. cremoris and *Leuconostoc mesenteroides* subsp. cremoris [7]. Mahewu is a popular South African indigenous refreshing non-alcoholic beverage produced by the fermentation of maize flour. Although traditionally the fermentation of mahewu is initiated by the addition of wheat flour, the industrial production can utilize both wheat flour and thermophilic *Lactobacillus culture* as a starter [8].

All above mentioned fermented products are typically characterized by consumers as having a pungent and rancid under-scent. With respect to the under-flavor, kefir, ryazhenka and amasi are characterized as buttery, and mahewu as waxy. Additionally, kefir and amasi possess a moderate acidic taste. While amasi does not have any pronounced main flavor, the main flavors of kefir and ryazhenka are alcoholic and roasty-sweet, respectively. Although the main flavor of fresh mahewu is extremely plain, it can obtain some additional flavor notes upon storage [4,8,9,10,11].

In the past decades, increasing consumers’ awareness about the numerous complex interactions between health and diet shifted the food market paradigm from the “food for nourish population” to the “food for health preservation and promotion” [12,13,14]. This shift forced food science to incorporate many modern methods and concepts from biochemistry, pharmacology, medicine and biotechnology [15,16]. Currently, many foodstuffs that were known and widely consumed for centuries are undergoing global scientific reevaluation and critical examination, and the study of traditional fermented foods form one of the most researched topics in this respect [17]. Among many potentially health-promoting properties of fermented food, the most relevant from the point of national dietary recommendations are their antioxidant, antihypertensive, anti-atherogenic and anti-thrombogenic properties [18]. Additionally, investigations of the volatile organic compounds (VOC) responsible for the characteristic aromas and flavors of fermented food form an important area of research, since products’ VOC profile has a strong influence on consumers’ preference and level of consumption [19,20]. Moreover, recent development of a concept (though controversial) regarding food (or flavor) pairing, which states “that ingredients sharing flavor compounds are more likely to taste well together than ingredients that do not”, promotes VOC analysis in as many products as possible to incorporate them into flavor networks [21,22].

Therefore, this study was undertaken to analytically characterize commercialized fermented beverages widely consumed in Russia (kefir and ryazhenka) and South Africa (amasi and mahewu) and to establish their potential health-promoting properties. For each product, the antioxidant capacity and ability to inhibit the angiotensin I converting enzyme (ACE) were assessed; for dairy beverages (kefir, ryazhenka and amasi), the profiles of fatty acids (FA) were measured and health-related FA nutritional indices were calculated. Additionally, the VOC profile of each product was determined. While for kefir several studies describing its antioxidant and antihypertensive properties, as well as FA profile and volatile organic compounds, have been previously published, to the best of our knowledge, currently, there are no such published data regarding ryazhenka, amasi and mahewu.

## 2. Materials and Methods

### 2.1. Studied Beverages

In the current work, the most widely consumed commercial fermented beverages were used: kefir and ryazhenka were purchased in the “Azbuka Vkusa” distribution network (Moscow, Russia), and amasi and mahewu were purchased in the distribution network of Durban (South Africa). The information contained in the labeling of beverages is shown in Table 1. For each product listed in Table 1 (three kefir products, two ryazhenka products, three amasi products and one mahewu product), three packs (samples) were purchased.

### 2.2. Measurments of pH, Antioxidant Capacity, ACE Inhibitory Activity and Degree of Proteolysis

The pH of the samples was determined using SevenEasy S20 pH meter (Mettler Toledo, Zurich, Switzerland) by the direct insertion of the electrode in the sample.

All samples were centrifuged at 10,000× *g* for 20 min at 4 °C. The supernatant was filtered through a 0.45 µm syringe filter and stored at −80 °C until further analysis.

The antioxidant capacity was determined with Oxygen Radical Absorbance Capacity (ORAC) assay according to [23]. In this assay, peroxyl radicals are generated directly in the reaction medium during the thermal decomposition (37 °C for 10 min) of the 2,2′-azobis (2-methylpropionamidine) dihydrochloride (AAPH, Sigma-Aldrich, St. Louis, MO, USA). The antioxidant activity was expressed as the amount of Trolox (Sigma-Aldrich, St. Louis, MO, USA) molar equivalents (µM TE).

The in vitro ACE inhibitory (ACE-I) was determined by their ability to inhibit conversion of o-Aminobenzoyl-Phe-Arg-Lys(dinitrophenyl)-Pro (Sigma-Aldrich, St. Louis, MO, USA) by ACE (Sigma-Aldrich, St. Louis, MO, USA) as described in [23]. The obtained data were normalized by the protein content determined using the Pierce BSA Protein Assay Kit (ThermoFisher, Rockford, IL, USA), and the half maximal inhibitory concentration (IC50) was expressed as mg of protein per ml.

The degree of proteolysis was assessed spectrophotometrically (at 340 nm) using the 2,4,6-trinitrobenzenesulfonic acid solution (TNBS, Sigma-Aldrich, St. Louis, MO, USA) method. The calibration curve was prepared using L-leucine (L-Leu) as a standard (0.1–2.0 mM). The results were expressed as L-Leu molar equivalents (mM (Leu)).

All measurements were performed at 37 °C in 96-well black microplates using Synergy 2 microplate photometer–fluorometer (BioTek, Winooski, VT, USA) equipped with an automatic thermostatic holder.

### 2.3. Fatty Acid Analysis

Fats were extracted from the samples according to Folch method, and equivalent volumes of extracts from the same product type (i.e., kefir, ryazhenka, amasi and mahewu) were pooled together. The extracted fatty acids were derivatized (i.e., fatty acid methyl esters were prepared) by acid-catalyzed transmethylation with 3 M methanolic HCl (Supelco, Bellefonte, USA), according to the manufacturer’s protocol. Chromatographic separation on an MDN-5 column (30 m × 0.25 mm; Supelco, Japan) was carried out using GC 2010 chromatograph (Shimadzu, Kyoto, Japan) equipped with a mass detector GCMS-QP 2010 in the regime of temperature gradient (initial hold at 70 °C for 1 min; heating up to 90 °C at a rate of 4 °C per min; heating up to 240 °C at a rate of 10 °C per min; hold at 240 °C for 4 min; heating up to 300 °C at a rate of 15 °C per min; final hold at 300 °C for 3 min) at the following temperatures: injector, 200 °C; interface, 210 °C; and detector, 200 °C. Helium with a flow of 1.0 mL·min^–1^ and a flow pressure of 1:20 was used as a carrier gas. The total analysis time was 32 min. Mass detection was carried out under the TIC registration mode in the mass acquisition range (*m/z*) from 45 to 400 (Supplementary Materials Figures S1–S3). The PUFA-2 (Supelco, Bellefonte, PE, USA), fatty acids of animal origin, were used as a standard. The relative intensities (further relative abundances) of fatty acids were obtained by normalization on the total intensity of the assigned peaks. All experiments were performed in triplicate.

FA were identified by comparing their experimental spectra with those from the National Institute of Standards and Technology (NIST/EPA/NIH Mass Spectral Database (NIST 11), Gaithersburg, MD, USA). Only the assignments of peaks with ≥90% confidence were considered reliable and reported here.

FA nutritional indices were calculated according to Chen et al. [24]

(1) $$PUFA/SFA=\frac{\sum PUFA}{\sum SFA}$$

(2) $$IA=\frac{C12:0+\left(4\times C14:0\right)+C16:0}{\sum MUFA+\sum PUFA}$$

(3) $$HPI=\frac{\sum MUFA+\sum PUFA}{C12:0+\left(4\times C14:0\right)+C16:0}$$

(4) $$IT=\frac{C14:0+C16:0+C18:0}{0.5\times \left[\sum MUFA+\sum PUFA\left(n-6\right)\right]}$$

(5) $$HH=\frac{C18:1+\sum PUFA}{C12:0+C14:0+C16:0}$$

(6) UI=1×(%monoenoics)+2×(%dienoics)+3×(%trienoics)+4×(%tetraenoics)

### 2.4. Volatile Organic Compounds Analysis

For the VOC analysis, equivalent volumes of products of the same type (i.e., kefir, ryazhenka, amasi and mahewu) were pooled together. The volatile compounds were extracted by solid phase micro extraction (SPME) and analyzed by gas chromatography coupled to mass spectrometry (GC–MS, GS 2010 gas chromatograph (Shimadzu, Kyoto, Japan) directly attached to a GCMS-QP 2010 mass spectrometer (Shimadzu, Kyoto, Japan)). SPME extractions were performed using 50/30 mm thick PDMS/DVB (polydimethylsiloxane/divinylbenzene) fibers (Supelco, Bellefonte, PA, USA) and 40 mL flasks fitted with mininert valves (Supelco, Bellefonte, PA, USA) that were inserted above the gas-phase bottle for extraction for 60 min at 40 °C. Desorption was conducted at 250 °C for 3 min. The Optima-1 column (25 m × 0.25 mm, Supelco, Japan), precalibrated with retention index standards (Sigma, St. Louis, MO, USA) of C8 and C32 aliphatic hydrocarbons, was initially held 2 min at 70 °C, then heated to 90 °C at a rate of 4 °C per min, to 160 °C at a rate of 10 °C per min, and finally heated to 280 °C at a rate of 20 °C per min, which was held for 5 min; at the following temperatures: injector, 230 °C; interface, 205 °C; detector, 200 °C. Helium with a flow rate of 0.7 mL·min^–1^ and a flow pressure of 1:20 was used as a carrier gas. The total analysis time was 25 min. Mass detection was carried out under the TIC registration mode in the mass acquisition range (*m/z*) from 45 to 450 (Supplementary Materials Figures S4–S7). The relative intensities (further relative abundances) of VOC were obtained by normalization on the total intensity of the assigned peaks. All experiments were performed in triplicate.

Volatile compounds were identified by comparing their experimental spectra with those from the National Institute of Standards and Technology (NIST/EPA/NIH Mass Spectral Database (NIST 11), Gaithersburg, MD, USA). Only the assignments of peaks with ≥90% confidence were considered reliable and reported here.

The data about flavors of individual compounds were extracted from FlavorDB [25]. For the concise descriptions of flavor, the flavor wheel developed by Foodpairing company was adopted [26]. For the graphical data representations “ggplot2” (donut charts) and “eulerr” (area-proportional Euler diagram) R packages were used. The color pallets were taken from RColorBrewer and Polychrome R packages.

### 2.5. Statistical Analysis

All statistical comparisons were firstly performed using one-way ANOVA omnibus F-Test. When a significant (*p* < 0.05) value of F-statistics was found, differences between means were evaluated using Tukey’s HSD (honestly significant difference) multiple comparison test (*p* < 0.05).

## 3. Results and Discussion

### 3.1. Antioxidant and Antihypertensive Properties

For all beverages, pH, antioxidant capacity and antihypertensive activity are shown in Table 2. The antioxidant capacity was measured against peroxyl radical using ORAC assay, and the antihypertensive activity by the beverages’ ability to inhibit ACE. Additionally, Table 2 includes data regarding the beverages’ degree of proteolysis measured as an amount of free amino groups and expressed as L-Leu equivalents. Since no significant differences in antioxidant capacity, ACE-I activity and degree of proteolysis were determined between beverages of the same type (i.e., kefir, ryazhenka, amasi and mahewu) but from different manufacturers, in the following text all data regarding the same beverage type were pulled together and reported as mean ± standard deviation.

The pH values of kefir, ryazhenka and amasi were in the range 4.4–4.5, while the pH value of mahewu was substantially lower and comprised approximately 3.5, which is consistent with previously reported data for these products [4,5,6].

Generally, the antioxidant capacity of the studied beverages decreased in a row: amasi > kefir = ryazhenka > mahewu, while ACE-I activity decreased (i.e., IC_50_ increased) in a row: mahewu > amasi > kefir = ryazhenka. At the same time, the degree of proteolysis decreased in a row: amasi > kefir > ryazhenka > mahewu. It is well known that antioxidant and ACE-I properties of fermented products are primarily attributed to bioactive peptides produced through the action of the cell envelope proteinases (CEPs) of starter cultures [27]. Since for dairy-based beverages both antioxidant capacity and ACE-I activity correlated with the degree of proteolysis, it can be proposed that kefir, ryazhenka and amasi starters produced almost similar bioactive peptides, and their amounts are primarily responsible for the observed overall activities.

In the case of mahewu, having the lowest degree of proteolysis, this beverage demonstrated the lowest antioxidant activity and the highest ACE-I activity among all studied beverages. This situation can be explained by the different source of bioactive peptides in this beverage. While in dairy-based fermented beverages the primary release source of bioactive peptides is caseins [28], in corn-based fermented beverages it is zein and glutelin [29]. The activities of bioactive peptides are determined by both their amino acid compositions and sequences, and the ACE-I peptides are more sequence-specific than antioxidant ones [30]. Hence, while the lowest antioxidant activity of mahewu is a result of the lowest amount of peptides in this beverage, the highest ACE-I activity suggests the presence of some peptides with very strong ability to perform competitive inhibition at the catalytic site of ACE.

Currently, a number of scientific studies have confirmed the probiotic potential and the health benefits of kefir. It has been shown that the regular consumption of kefir improves digestion, leads to a decrease in blood glucose in patients with type 2 diabetes, improves lipid profile, lowers blood cholesterol, normalizes blood pressure, leads to a decrease in the concentration of free radicals in the blood and has a number of other health-promoting effects [31]. In contrast, studies confirming the health-promoting effects of ryazhenka, amasi and mahewu are currently absent; however, due to the presence of probiotic strains of lactic acid bacteria in the starter cultures, these products are defined as beneficial to health [4,32,33].

In the presented study it was demonstrated that ryazhenka and mahewu possess antioxidant capacity comparable to kefir, while the antioxidant capacity of amasi was significantly higher. As for the ACE-I activity, in ryazhenka it was slightly lower than in kefir, and in amasi and mahewu it was approximately 1.6 and 3 times higher, respectively. The obtained in vitro results allow hypothesizing that ryazhenka, amasi and mahewu will possess antioxidant and hypotensive properties in vivo; however, this requires further confirmation. The high values of ACE-I activity in mahewu also make this corn-based product a promising source for the discovery of new biologically active antihypertensive peptides.

### 3.2. Profile of Fatty Acids

The qualitative composition and relative abundances (percentage from total FA) of FA in dairy-based beverages is shown in Table 3. Unfortunately, the low total fat content of mahewu (less than 1%, Table 1) did not allow a reliable extraction process to be performed. In total, in all beverages, 31 different FA were detected, 15 of which belonged to the group of saturated fatty acids (SFA), five to monounsaturated fatty acids (MUFA), four to polyunsaturated fatty acids (PUFA, all “*n*-6”), four to branched chain fatty acids (BCFA) and three 2-hydroxy fatty acids (2OH-FA). Qualitatively, the greatest number of different FA were detected in kefir (15—SFA, five—MUFA, four—PUFA, three—BCFA and three—2OH-FA), followed by ryazhenka (15—SFA, five—MUFA, three—PUFA, two–BCFA and one—2OH-FA) and amasi (11—SFA, four—MUFA, one—PUFA and three—BCFA). All FA detected in ryazhenka and amasi were also present in kefir; the exception was one BCFA (anteiso-C17:0) uniquely presented in amasi. In terms of overall FA contents, in amasi the proportion of SFA and BCFA (65% and 1.8%, respectively) was significantly higher than in kefir (63 and 1.2%, respectively) and ryazhenka (64 and 1.2%, respectively); the proportion of PUFA (2.0%) was significantly lower than in kefir (5.5%) and ryazhenka (5.0%); the proportion of MUFA was statistically the same (30–31%).

It is well known that the consumption of certain dietary fats, which are generally FA, may exert either positive or negative effects on human health [34,35,36]. To evaluate the potential role of FA in the treatment and prevention of diseases, many nutritional indices for assessing FA content of food have been developed over time. Currently, the most frequently used for the evaluation of different food FA nutritional indices are [24]: PUFA/SFA—ratio of total PUFA to total SFA; IA—index of atherogenicity; HPI—health-promoting index (which is the reciprocal of IA and mainly used in research on dairy products); IT—index of thrombogenicity; HH—hypocholesterolemic/hypercholesterolemic ratio; UI—unsaturation index. The calculation of mentioned indices for the studied dairy beverages is presented in Table 4. Generally, all of the calculated indices were in the range previously reported for different yogurts [24]. Interestingly, all FA indices of kefir calculated in this study were significantly better (i.e., higher PUFA/SFA, HPI, HH and UI; lower IA and IT) than those previously reported for milk fermented with different kefir grains [37]. This can be possibly explained by different kefir preparation processes: while in Russia commercial kefir is prepared using intermediate starter (“mother culture”) [4], many scientific articles utilize “traditional” fermentation by kefir grains themselves [38]. The comparison of studied dairy beverages in terms of the mentioned FA nutritional indices clearly showed that these indices for kefir and ryazhenka were similar and significantly better than for amasi.

While previously mentioned indices are calculated using only SFA, MUFA and PUFA in one way or another, they totally missed two important classes of FA—BCFA and 2OH-FA. While the dietary implication of 2OH-FA is still largely unknown [39,40], BCFA have recently been proposed as bioactive molecules with pro-health benefits [41]. Moreover, the human body can derive only a limited amount of BCFAs from dietary branched chain amino acids, and dietary fats remain the most important source of BCFA within the body [42]. The determined contents of BCFA for kefir and ryazhenka were similar (1.17–1.19%) but, in contrast to the FA nutritional indices, they were significantly worse (i.e., lower) than for amasi (1.8%).

### 3.3. Volatile Organic Compounds

**Figure 1:**
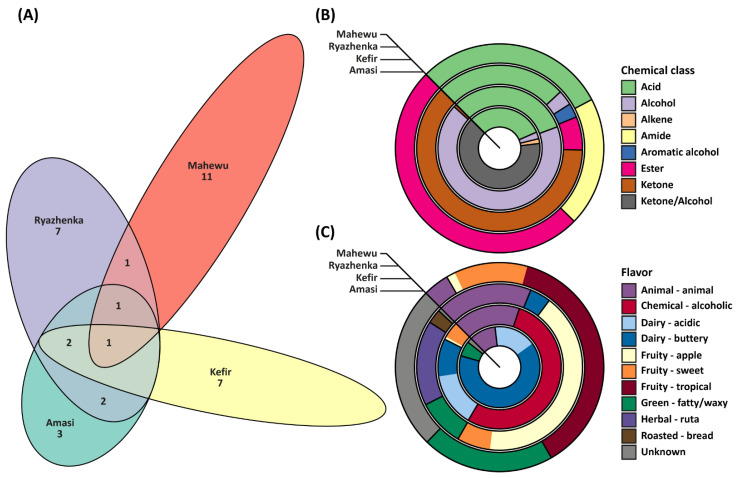
Summary of volatile organic compounds determined in kefir, ryazhenka, amasi and mahewu: (**A**) Area-proportional Euler diagram (the numbers represent the powers of corresponding sets); (**B**) Chemical classes of compounds; (**C**) Flavor classes of compounds.

For all studied fermented beverages, GC-MS analysis allowed the presence of 35 different VOC to be determined (Table 5). The greatest number of VOC were determined for ryazhenka and mahewu, 14 compounds for each, while for kefir and amasi 10 and 9 compounds were determined, respectively. Generally, the number of compounds shared by several beverages were small compared to the number of beverage-specific compounds (Figure 1A). Only one compound, hexanoic acid, was detected in all beverages; two compounds, octanoic acid and decanoic acid, were common for all dairy-based beverages (i.e., kefir, ryazhenka and amasi); two compounds, 1-hexadecanol and stearic acid, were common for ryazhenka and amasi; one compound, palmitic acid, was common for mahewu, ryazhenka and amasi; one compound, ethyl methyl carbonate, was common for mahewu and ryazhenka. In terms of beverage-specific compounds, the greatest number, 11, was determined in mahewu, followed by kefir, ryazhenka and amasi, which had seven, seven and three beverage-specific compounds, respectively. The relative abundance of beverage-specific VOC in kefir, ryazhenka, amasi and mahewu comprised 82, 69, 83 and 77%, respectively.

For each beverage, the distribution of VOC according to their chemical classes is presented in Figure 1B. All products contained almost an equal relative amount of acid compounds, approximately 30%. Alcohol compounds were detected only in dairy-based beverages (67% in kefir, 3% in ryazhenka and 3% in amasi); ketone compounds were detected only in kefir (1%) and ryazhenka (62%); ester compounds were detected only in ryazhenka (6%) and mahewu (50%). For the beverage-specific classes of compounds: aromatic alcohol (furfuryl alcohol) was detected only in ryazhenka (3%); ketone/alcohol (acetoin) was detected only in amasi (64%); amide (lactamide) was detected only in mahewu (20%).

For each beverage, all determined VOC were classified according to their flavors, and the overall beverages’ theoretical (predicted) flavor profiles are presented in Figure 1C. In general, the theoretical flavor profiles can be very different from the actual flavor because of humans’ multisensory flavor perception [20]. However, in the case of the studied beverages, the theoretical flavor profiles and actual flavors were in accordance, which allowed for hypothesizing about the contribution of the detected VOC to the overall flavor.

It can be proposed that in all products, all of the animal scents can be attributed to the presence of one or more of the following medium-chain fatty acids: hexanoic, octanoic, decanoic and (E)-hex-2-enoic acid. Previously, the presence of these acids was established for several dairy and cereal-based fermented foods and beverages [43,44,45]. Moreover, the presence of hexanoic acid was linked with the pungent, rancid, flowery flavor of yogurt by many researchers [19,46].

While the buttery under-flavor of kefir can be explained by the presence of 2,3-butanediol (10%), in ryazhenka and amasi, this under-flavor is probably produced by the presence of butyric (or butanoic) acid (3%) and acetoin (64%), respectively. Although the relative abundance of butyric acid in ryazhenka was low, previously, this acid, along with hexanoic acid, was determined as one of the most flavor-producing components for the fermented baked milk (a ryazhenka-like product) [47].

The acidic flavor of kefir and amasi can be attributed to acetic acid (14%) and 3-hydroxypropionic acid (17%), respectively. The main alcoholic flavor of kefir can be attributed to 3-methyl-1-butanol (36%) and ethanol (16%). Both of these VOC are well known flavor-forming compounds in kefir. While ethanol is produced by kefir grains as a result of sugar fermentation by yeasts, 3-methyl-1-butanol is formed from isoleucine through deamination and decarboxylation reactions [48]. Previously, it was shown that the VOC profiles of various kefir beverages obtained from different milk (e.g., goat and cow) using kefir grains with different microbial composition can vary greatly [49,50]. Nevertheless, compounds such as 3-methyl-1-butanol, ethanol, acetic acid, hexanoic acid, octanoic acid, decanoic acid and benzoic acid were determined as common kefirs’ VOC, while the profile of ketones, aldehydes, esters and other alcohols varied greatly depending on the origin of the kefir grains.

In ryazhenka, the presence of furfuryl alcohol (possessing a distinctive burnt caramel flavor) as well as the abundance of ketones, can be attributed to the baking process that initiates the Maillard reaction in milk [51]. It should be noted that, except for furfuryl alcohol, there were no other products of the Maillard reaction (i.e., 2,5-dimethyl-4-hydroxy-3(2H)-furanone and 2-methylbutyric acid/2-methyl-3-(methyldithio)furan) with a caramel flavor detected in ryazhenka, in contrast to the ryazhenka-like product [47]. This can be explained by the low volatility of such compounds, and the necessity for more sophisticated experimental procedures to enhance their extraction yield [47].

While for ryazhenka the fruity flavor can be explained by the abundance of ketones, for the mahewu, this flavor is attributed to esters. In the group of esters, one should note the dominant content of acetic acid pentyl ester (pentyl acetate, 20%), which, along with esters of butyric and isobutyric acids, pelargonic acid ethyl ester (ethyl nonanoate) and caproic acid allyl ether (prop-2-enyl hexanoate), gives the product a pleasant, fresh, banana-resembling fruity aroma. The presence of esters in mahewu is most probably related to the metabolic activity of yeasts. Although for mahewu yeasts are a non-starter culture, they are always present in the final product; in mahewu, an acceptable yeast concentration was previously reported as 10^6^ CFU·mL^−1^ [8]. Moreover, water absorption by the corn meal decreases water activity in the medium, which, in turn, promotes esterification reactions [52].

## 4. Conclusions

Current increased consumers’ awareness toward “healthy food”, as well as the necessity to form national dietary recommendations in order to improve population health, demands the reevaluation of many century-known products using modern analytical technics. In this respect, the study of fermented food traditionally consumed in large quantities is of paramount importance. The current investigation of the health-related properties of commercialized kefir, ryazhenka, amasi and mahewu demonstrated that: (1) generally, dairy-based fermented beverages (kefir, ryazhenka, amasi) outperform cereal-based (mahewu) in terms of antioxidant capacity; however, mahewu demonstrated superior ACE-I activity; (2) among dairy-based beverages, although kefir and ryazhenka were better in terms of FA nutritional indices, both of these products contained significantly lower amounts of BCFA than amasi. For all beverages, the VOC profile was in accordance with consumers’ flavor perception, which allows of the use of food pairing concepts (i.e., identifying which foods go well together from a flavor standpoint) to compose rations with these products. The obtained data contribute to the knowledge regarding traditional fermented beverages and set new perspectives on these beverages in terms of national dietary recommendations.

## Figures and Tables

**Table 1:** Information from the labels of fermented beverages used in this study.

Product Name, Type of Packaging, Net Weight	Manufacturer, Address	Nutritional Information per 100 g of Product	Ingredients:
«Ruzskyi Kefir» fat 3.2–4.0%. Hermetically packed in a Pure-Pak, m = 1000 g	JSC “RUZKE MILK”, Russia, Moscow Region.	Fat—3.8 g Protein—3.0 g Carbohydrate—4.0 g Energy—from 238 to 268 kJ/ from 57 to 64 kcal	Whole milk, culture on kefir grains. The amount of yeast at the end of the shelf life is at least 10^4^ CFU·g^–1^. The number of lactic acid microorganisms is not less than 10^7^ CFU·g^–1^.
Kefir «Asenievskaya ferma» fat 3.2%. Hermetically packed in a polypropylene bottle, m = 450 g	Agricultural production cooperative “Agricultural artel (collective farm) “Pervomaisky”. Russia, Kaluga region.	Fat—3.2 g Protein—3.0 g Carbohydrate—4.0 g Energy—238 kJ/57 kcal	Normalized milk, culture on kefir grains. The number of lactic acid producing microorganisms is not less than 10^7^ CFU·g^–1^. The amount of yeast at the end of the shelf life is at least 10^4^ CFU·g^–1^.
Kefir «Molochnaya kultura» fat from 3.5 to 4.5%. Hermetically packed in a polypropylene glass, m = 500 g	Milk Culture LLC, Russia, Leningrad Region.	Fat—4.1 g Protein—3.0 g Carbohydrate—4.0 g Energy—60–70 kcal/ 250–290 kJ	Whole milk, culture on kefir grains. The number of lactic acid producing microorganisms is not less than 1 × 10^7^ CFU·g^–1^. The amount of yeast at the end of the shelf life is at least 1 × 10^4^ CFU·g^–1^.
Ryazhenka «Ruzskaya» fat 2.5%. Hermetically packed in a Pure-Pak. m = 330 g	JSC “RUZKE MILK”, Russia, Moscow Region.	Fat—2.5 g Protein—3.0 g Carbohydrate—4.0 g Energy—from 211 kJ/50 kcal	Normalized baked milk, fermentation of lactic acid producing microorganisms. The number of lactic acid microorganisms is not less than 1 × 10^7^ CFU·g^–1^.
Ryazhenka «Molochnaya kultura» fat from 3.5 to 4.5%. Hermetically packed in a polypropylene glass, m = 500 g	Milk Culture LLC, Russia, Leningrad Region.	Fat—3.8 gProtein—3.0 g Carbohydrate—4.1 g Energy—60–70 kcal/ 250–290 kJ	Whole milk, fermentation of lactic acid producing microorganisms. The number of lactic acid microorganisms is not less 1 × 10^7^ CFU·g^–1^.
«Full cream MAAS PASTEURISED» fat 3.7%. Hermetically packed in a polypropylene bottle, m = 500 g	Specially packaged for Pick n Pay Ltd. «PnP», South Africa, Kensington.	Fat—3.7 g Protein—3.3 g Carbohydrate—5.0 g Energy—270 kJ	Full Cream milk, Starter Culture.
Full cream MAAS «AMASI OTHANDO» fat 3.3%. Hermetically packed in a Pure-Pak, m = 500 g	Clover S.A. (PTY) LTD Clover Park, South Africa, Roodepoort.	Fat—3.3 g Protein—3.3 g Carbohydrate—4.0 g Energy—241 kJ	Milk and/or Recombined Milk, Culture.
Full cream MAAS «INKOMAZI Rich and Creamy» fat 3.4%. Hermetically packed in a cardboard bag (Nampak Liquid Cartons), m = 500 g	Manufactured for Danone Southern Africa (PTY) LTD, 99 Skew Road, Boksburg North, 1459, South Africa	Fat—3.4 g Protein—3.2 g Carbohydrate—5.0 g Energy—265 kJ	Full fat milk, MAAS cultures.
«MNANDI Amahewu Creamy flavour» fat < 0.1%. Hermetically packed in a Pure-Pak, m = 1 L	«RcL Foods», South Africa	Fat < 0.1 g Protein—0.7 g Carbohydrate—6.0 g Energy—122 kJ	Water, Maize (7%), Lactic Acid producing culture, Sugar, Flavouring, Preservative (Potassium Sorbate), Non- nutritive sweeteners (Sodium cyclamate, Sodium saccharin, Aspartame), Contains Phenylalanine.

**Table 2:** Antioxidant capacity, ACE-I activity, pH and degree of proteolysis.

Product	pH	ORAC, µmol(TE)·mL^−1^	ACE-I Activity (IC_50_), mg(Protein)·mL^−1^	mM(Leu)
Kefir	4.44 ± 0.03	430 ^a^ ± 92	2.75 ^a^ ± 0.61	5.71 ^a^ ± 0.56
Ryazhenka	4.46 ± 0.06	414 ^a^ ± 43	3.94 ^a^ ± 1.92	3.72 ^b^ ± 0.67
Amasi	4.47 ± 0.04	682 ^b^ ± 82	1.72 ^b^ ± 0.39	8.32 ^c^ ± 0.72
Mahewu	3.54 ± 0.02	326 ^c^ ± 52	0.92 ^c^ ± 0.67	0.68 ^d^ ± 0.12

TE—Trolox equivalents; IC_50_— the half maximal inhibitory concentration; Leu—L-Leu equivalents. ^a,b,c,d^ Means within the same column with different superscripts are significantly different (*p* < 0.05).

**Table 3:** Fatty acids (FA) composition of kefir, ryazhenka and amasi.

Fatty Acid		Relative Abundance, %					
Name	Abbreviation	Kefir		Ryazhenka		Amasi	
		Mean	SD	Mean	SD	Mean	SD
Saturated Fatty Acids (SFA)							
Butanoic acid	C4:0	1.82 ^a^	0.054	1.64 ^b^	0.092	ND	-
Pentanoic acid	C5:0	0.04	0.011	0.03	0.009	ND	-
Hexanoic acid	C6:0	1.77	0.126	1.65	1.002	1.81	0.254
Heptanoic acid	C7:0	0.05	0.017	0.04	0.010	ND	-
Octanoic acid	C8:0	1.46 ^a^	0.025	1.30 ^b^	0.102	1.49 ^a^	0.132
Nonanoic acid	C9:0	0.07	0.008	0.05	0.003	ND	-
Decanoic acid	C10:0	3.63 ^a^	0.074	3.26 ^b^	0.021	4.00 ^c^	0.082
Undecanoic acid	C11:0	0.13	0.014	0.09	0.012	0.08	0.008
Dodecanoic acid	C12:0	4.41 ^a^	0.220	3.91 ^b^	0.081	4.07 ^c^	0.051
Tridecanoic acid	C13:0	0.16	0.022	0.14	0.021	0.09	0.030
Tetradecanoic acid	C14:0	10.48	0.682	10.84	0.321	10.38	0.260
Pentadecanoic acid	C15:0	1.50 ^a^	0.041	1.61 ^b^	0.021	1.22 ^c^	0.018
Hexadecanoic acid	C16:0	25.07 ^a^	0.173	24.32 ^b^	0.210	25.66 ^c^	0.050
Heptadecanoic acid	C17:0	0.63	0.010	1.24	0.021	0.52	0.010
Octadecanoic acid	C18:0	11.90 ^a^	0.193	13.53 ^b^	0.224	15.83 ^c^	0.150
Total SFA		63.11 ^a^	0.780	63.65 ^a^	1.109	65.15 ^b^	0.430
Monounsaturated fatty acids (MUFA)							
4-Decenoic acid	C10:1 (*n* − 6)	0.38	0.021	0.34	0.008	0.35	0.006
9-Tetradecenoic acid	C14:1 (*n* − 5)	1.21	0.012	1.16	0.026	0.85	0.041
9-Hexadecenoic acid	C16:1 (*n* − 7)	1.92	0.081	1.72	0.102	1.28	0.026
9-Octadecenoic acid	C18:1 (*n* − 9)	20.45 ^a^	0.410	21.72 ^b^	0.320	28.53 ^c^	0.970
12-Octadecenoic acid	C18:1 (*n* − 6)	6.06	1.021	5.21	0.524	ND	-
Total MUFA		30.02	1.103	30.15	0.623	31.02	0.971
Polyunsaturated fatty acids (PUFA)							
9,12-Octadecadienoic acid	C18:2 (*n* − 6)	4.04 ^a^	0.140	3.50 ^b^	0.250	2.04 ^c^	0.134
10,12-Octadecadienoic acid	C18:2 (*n* − 6)	1.06 ^a^	0.014	1.29 ^b^	0.008	ND	-
8,11,14-Eicosatrienoic acid	C20:3 (*n* − 6)	0.23	0.023	ND	-	ND	-
5,8,11,14-Eicosatetraenoic acid	C20:4 (*n* − 6)	0.19	0.025	0.16	0.016	ND	-
Total PUFA		5.53 ^a^	0.145	4.96 ^b^	0.251	2.04 ^c^	0.134
Branched chain fatty acids (BCFA)							
Tridecanoic acid, 12-methyl	iso-C14:0	0.26 ^a^	0.021	0.33 ^b^	0.024	ND	-
Tetradecanoic acid, 12-methyl	anteiso-C15:0	0.45 ^a^	0.010	0.53 ^b^	0.016	0.59 ^c^	0.023
14-methylhexadecanoic acid	anteiso-C17:0	ND	-	ND	-	0.57	0.071
15-methylhexadecanoic acid	iso-C17:0	0.45 ^a^	0.010	0.34 ^b^	0.016	0.62 ^c^	0.052
Total BCFA		1.17 ^a^	0.025	1.19 ^a^	0.033	1.79 ^b^	0.091
2-hydroxy fatty acids (2OH-FA)							
Hexanoic acid, 2-hydroxy	2OH-C6:0	0.08 ^a^	0.007	0.05 ^b^	0.002	ND	-
Butyric acid, 2-hydroxy-3-methyl	2OH-iso-C5:0	0.03	0.001	ND	-	ND	-
Pentanoic acid, 2-hydroxy-3-methyl	2OH-anteiso-C6:0	0.06	0.005	ND	-	ND	-
Total 2OH-FA		0.17 ^a^	0.009	0.05 ^b^	0.002	ND	-

ND: not detected; SD: standard deviation. ^a,b,c^ Means within the same row with different superscripts are significantly different (*p* < 0.05).

**Table 4:** Fatty acid (FA) nutritional indices of kefir, ryazhenka and amasi.

Index	Kefir		Ryazhenka		Amasi	
	Mean	SD	Mean	SD	Mean	SD
PUFA/SFA	0.088 ^a^	0.003	0.078 ^a^	0.004	0.031 ^b^	0.002
IA	2.008 ^a^	0.021	2.039 ^a^	0.011	2.155 ^b^	0.008
HPI	0.498 ^a^	0.005	0.490 ^a^	0.003	0.464 ^b^	0.002
IT	2.670 ^a^	0.172	2.774 ^a^	0.109	3.138 ^b^	0.187
HH	0.802 ^a^	0.031	0.816 ^a^	0.019	0.762 ^b^	0.025
UI	41.686 ^a^	1.113	40.391 ^a^	0.672	35.096 ^b^	0.980

SD: standard deviation; ^a,b^ Means within the same row with different superscripts are significantly different (*p* < 0.05).

**Table 5:** Volatile organic compounds determined in kefir, ryazhenka, amasi and mahewu.

IUPAC Name	Chemical Class	Concise Flavor Description	Relative Abundance, %
Kefir			
3-methylbutan-1-ol	Alcohol	Chemical—alcoholic	35.75
ethanol	Alcohol	Chemical—alcoholic	16.41
acetic acid	Acid	Dairy—acidic	14.11
butane-2,3-diol	Alcohol	Dairy—buttery	9.87
hexanoic acid	Acid	Animal—animal	8.89
octanoic acid	Acid	Animal—animal	7.24
2,7-dimethyloctane-4,5-diol	Alcohol	Fruity—sweet	4.31
decanoic acid	Acid	Animal—animal	1.59
hexan-1-ol	Alcohol	Chemical—alcoholic	1.11
octan-2-one	Ketone	Fruity—apple	0.72
Ryazhenka			
heptan-2-one	Ketone	Fruity—apple	41.64
nonan-2-one	Ketone	Herbal—ruta	16.55
hexanoic acid	Acid	Animal—animal	11.23
ethyl methyl carbonate	Ester	Fruity—sweet	6.49
octanoic acid	Acid	Animal—animal	5.57
undecan-2-one	Ketone	Green—fatty/waxy	3.33
butanoic acid	Acid	Dairy—buttery	3.17
hexadecan-1-ol	Alcohol	Green—fatty/waxy	2.95
furan-2-ylmethanol	Aromatic alcohol	Roasted—bread	2.77
octadecanoic acid	Acid	Green—Fatty/Waxy	2.48
decanoic acid	Acid	Animal—animal	1.86
nonanoic acid	Acid	Dairy—buttery	0.91
tetradecan-2-one	Ketone	Unknown	0.65
hexadecanoic acid	Acid	Green—fatty/waxy	0.39
Amasi			
3-hydroxybutan-2-one (acetoin)	Ketone/Alcohol	Dairy—buttery	64.44
3-hydroxypropanoic acid	Acid	Dairy—acidic	16.90
hexanoic acid	Acid	Animal—animal	6.76
octanoic acid	Acid	Animal—animal	3.34
hexadecan-1-ol	Alcohol	Green—fatty/waxy	2.74
octadecanoic acid	Acid	Green—Fatty/Waxy	2.45
dec-1-ene	Alkene	Unknown	1.90
hexadecanoic acid	Acid	Green—fatty/waxy	0.89
decanoic acid	Acid	Animal—animal	0.59
Mahewu			
pentyl acetate	Ester	Fruity—tropical	20.20
2-hydroxypropanamide	Amide	Unknown	20.04
hexadecanoic acid	Acid	Green—fatty/waxy	15.16
prop-2-enyl hexanoate	Ester	Fruity—tropical	6.85
ethyl methyl carbonate	Ester	Fruity—sweet	5.95
pentyl 2-methylpropanoate	Ester	Fruity—sweet	5.44
tetradecanoic acid	Acid	Green–fatty/waxy	5.36
(E)-octadec-11-enoic acid	Acid	Unknown	5.21
pentyl butanoate	Ester	Fruity—tropical	4.64
ethyl butanoate	Ester	Fruity—tropical	2.98
ethyl nonanoate	Ester	Fruity—tropical	2.76
hexanoic acid	Acid	Animal—animal	2.27
(E)-hex-2-enoic acid	Acid	Animal—animal	1.77
pentyl 2-methylbutanoate	Ester	Fruity—apple	1.38

## Data Availability

The data presented in this study are available in the article and supplementary materials.

## Supplementary Materials

The following are available online at https://www.mdpi.com/article/10.3390/foods10123082/s1, Figure S1: GS-MS total ion chromatogram (TIC)—Fatty acids composition of kefir, Figure S2: GS-MS total ion chromatogram (TIC)—Fatty acids composition of ryazhenka, Figure S3: GS-MS total ion chromatogram (TIC)—Fatty acids composition of amasi, Figure S4: GS-MS total ion chromatogram (TIC)—Volatile organic compounds of kefir, Figure S5: GS-MS total ion chromatogram (TIC)—Volatile organic compounds of ryazhenka, Figure S6: GS-MS total ion chromatogram (TIC)—Volatile organic compounds of amasi, Figure S7: GS-MS total ion chromatogram (TIC)—Volatile organic compounds of mahewu.
