# Supplementary material for: Analytical Characterization of the Widely Consumed Commercialized Fermented Beverages from Russia (Kefir and Ryazhenka) and South Africa (Amasi and Mahewu): Potential Functional Properties and Profiles of Volatile Organic Compounds
Source: Foods. 2021 Dec 11;10(12):3082. doi: 10.3390/foods10123082 (PMC8701341; doi:10.3390/foods10123082)
Supplement: Supplementary file 1 [file foods-10-03082-s001.zip › foods-1493381-supplementary.pdf]

Supplementary Materials

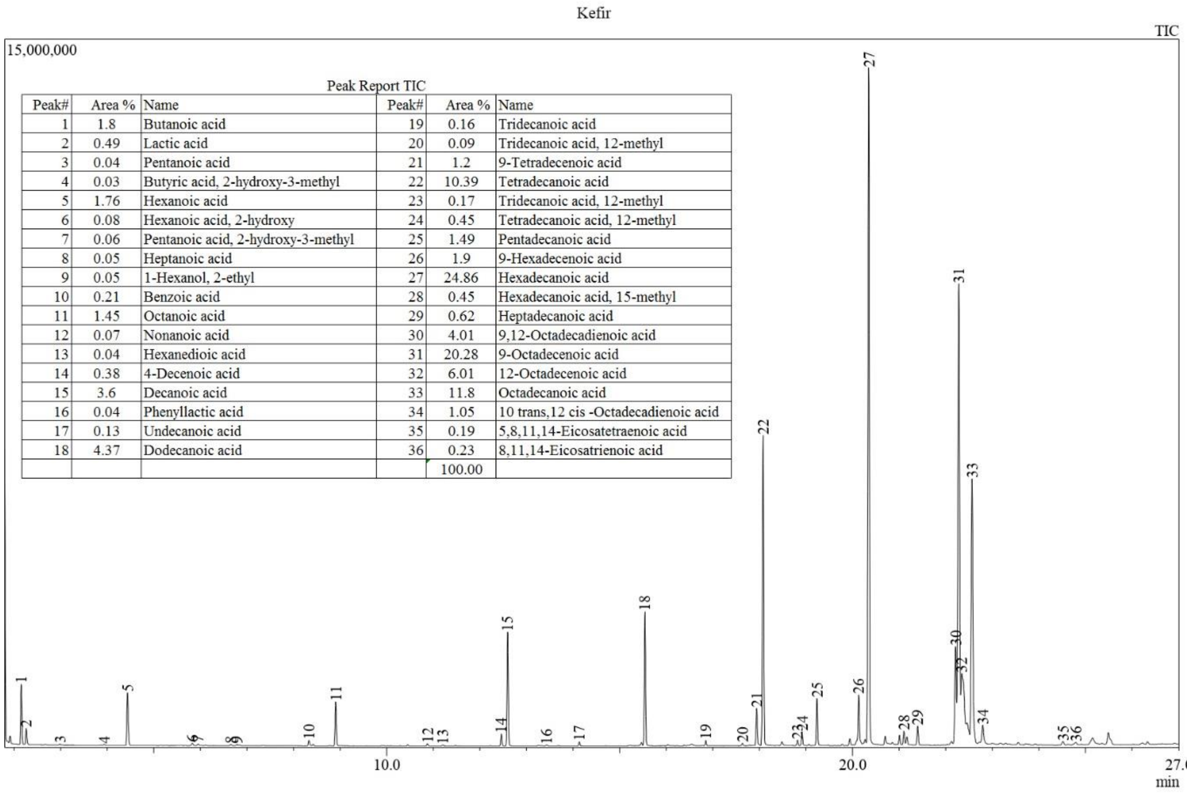

Peak#	Area %	Name	Peak#	Area %	Name
1	1.8	Butanoic acid	19	0.16	Tridecanoic acid
2	0.49	Lactic acid	20	0.09	Tridecanoic acid, 12-methyl
3	0.04	Pentanoic acid	21	1.2	9-Tetradecenoic acid
4	0.03	Butyric acid, 2-hydroxy-3-methyl	22	10.39	Tetradecanoic acid
5	1.76	Hexanoic acid	23	0.17	Tridecanoic acid, 12-methyl
6	0.08	Hexanoic acid, 2-hydroxy	24	0.45	Tetradecanoic acid, 12-methyl
7	0.06	Pentanoic acid, 2-hydroxy-3-methyl	25	1.49	Pentadecanoic acid
8	0.05	Heptanoic acid	26	1.9	9-Hexadecenoic acid
9	0.05	1-Hexanol, 2-ethyl	27	24.86	Hexadecanoic acid
10	0.21	Benzoic acid	28	0.45	Hexadecanoic acid, 15-methyl
11	1.45	Octanoic acid	29	0.62	Heptadecanoic acid
12	0.07	Nonanoic acid	30	4.01	9,12-Octadecadienoic acid
13	0.04	Hexanedioic acid	31	20.28	9-Octadecenoic acid
14	0.38	4-Decenoic acid	32	6.01	12-Octadecenoic acid
15	3.6	Decanoic acid	33	11.8	Octadecanoic acid
16	0.04	Phenyllactic acid	34	1.05	10 trans,12 cis -Octadecadienoic acid
17	0.13	Undecanoic acid	35	0.19	5,8,11,14-Eicosatetraenoic acid
18	4.37	Dodecanoic acid	36	0.23	8,11,14-Eicosatrienoic acid
				100.00	

Figure S1. GC-MS total ion chromatogram (TIC) – Fatty acids composition of kefir.

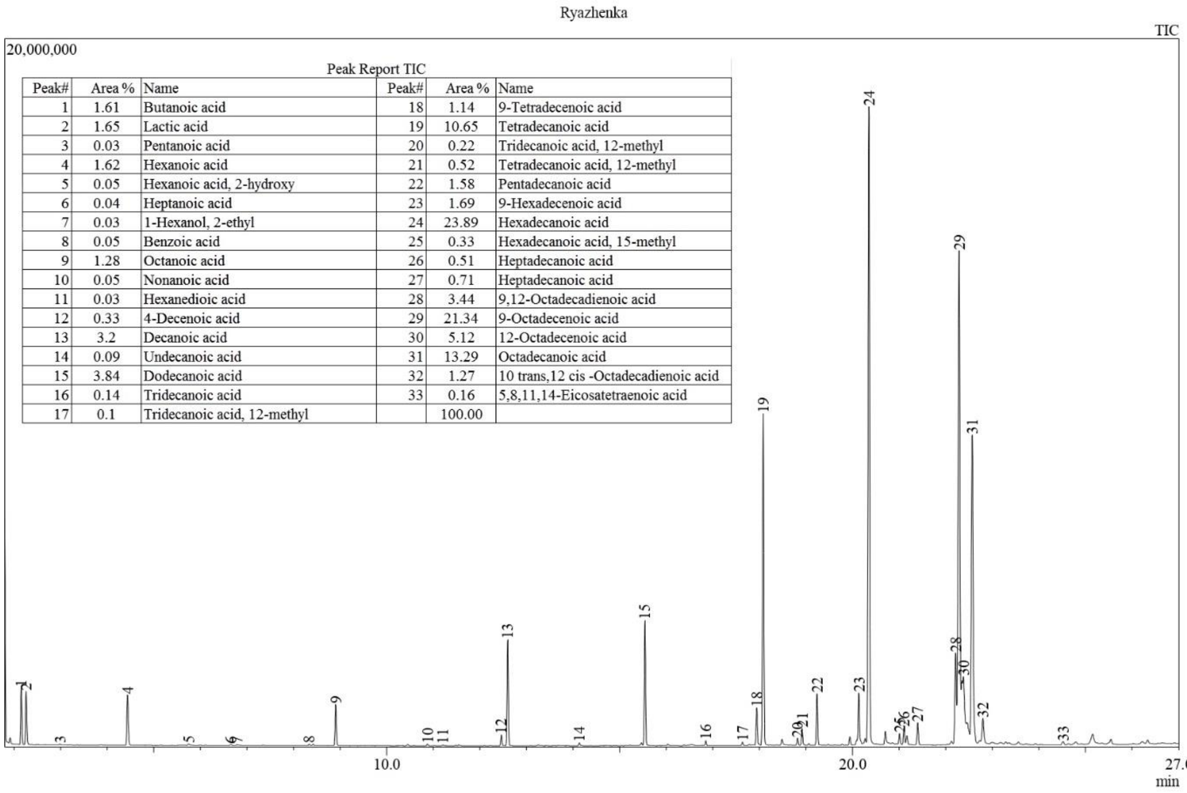

Peak#	Area %	Name	Peak#	Area %	Name
1	1.61	Butanoic acid	18	1.14	9-Tetradecenoic acid
2	1.65	Lactic acid	19	10.65	Tetradecanoic acid
3	0.03	Pentanoic acid	20	0.22	Tridecanoic acid, 12-methyl
4	1.62	Hexanoic acid	21	0.52	Tetradecanoic acid, 12-methyl
5	0.05	Hexanoic acid, 2-hydroxy	22	1.58	Pentadecanoic acid
6	0.04	Heptanoic acid	23	1.69	9-Hexadecenoic acid
7	0.03	1-Hexanol, 2-ethyl	24	23.89	Hexadecanoic acid
8	0.05	Benzoic acid	25	0.33	Hexadecanoic acid, 15-methyl
9	1.28	Octanoic acid	26	0.51	Heptadecanoic acid
10	0.05	Nonanoic acid	27	0.71	Heptadecanoic acid
11	0.03	Hexanedioic acid	28	3.44	9,12-Octadecadienoic acid
12	0.33	4-Decenoic acid	29	21.34	9-Octadecenoic acid
13	3.2	Decanoic acid	30	5.12	12-Octadecenoic acid
14	0.09	Undecanoic acid	31	13.29	Octadecanoic acid
15	3.84	Dodecanoic acid	32	1.27	10 trans,12 cis -Octadecadienoic acid
16	0.14	Tridecanoic acid	33	0.16	5,8,11,14-Eicosatetraenoic acid
17	0.1	Tridecanoic acid, 12-methyl		100.00	

Figure S2. GC-MS total ion chromatogram (TIC) – Fatty acids composition of ryazhenka.

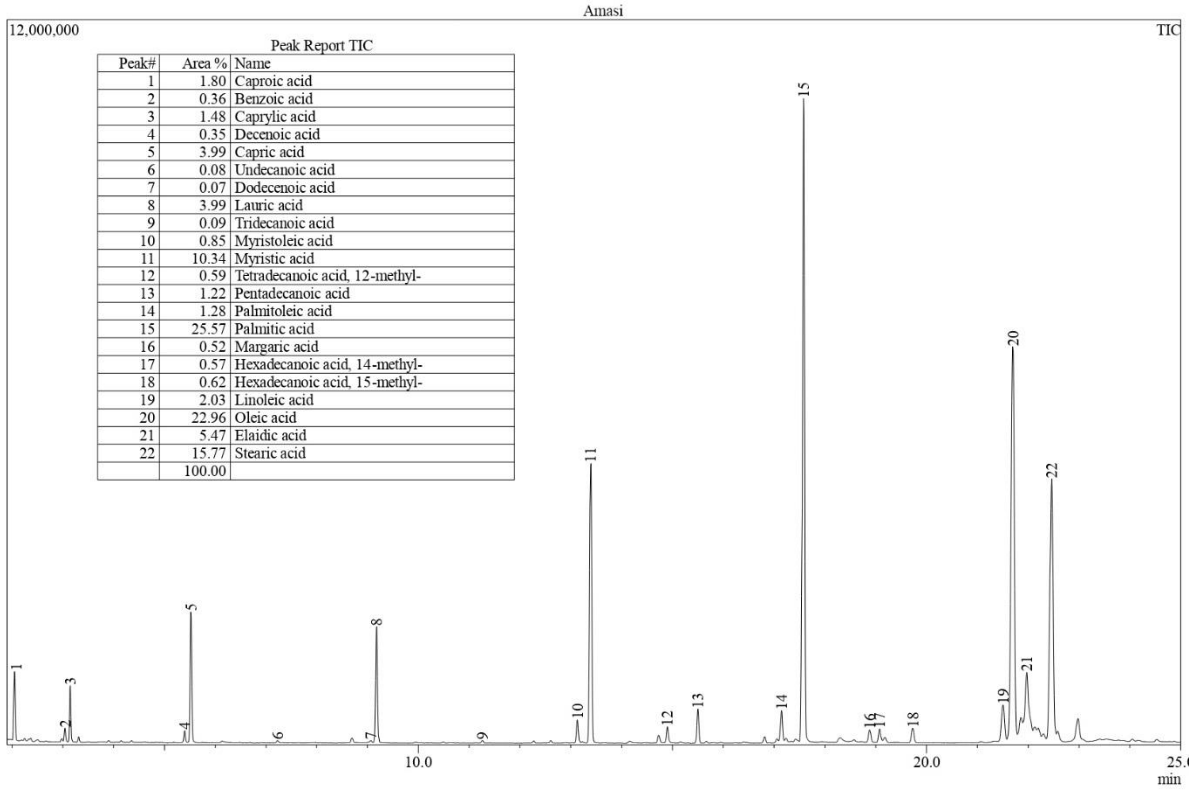

Peak#	Area %	Name
1	1.80	Caproic acid
2	0.36	Benzoic acid
3	1.48	Caprylic acid
4	0.35	Decenoic acid
5	3.99	Capric acid
6	0.08	Undecanoic acid
7	0.07	Dodecenoic acid
8	3.99	Lauric acid
9	0.09	Tridecanoic acid
10	0.85	Myristoleic acid
11	10.34	Myristic acid
12	0.59	Tetradecanoic acid, 12-methyl-
13	1.22	Pentadecanoic acid
14	1.28	Palmitoleic acid
15	25.57	Palmitic acid
16	0.52	Margaric acid
17	0.57	Hexadecanoic acid, 14-methyl-
18	0.62	Hexadecanoic acid, 15-methyl-
19	2.03	Linoleic acid
20	22.96	Oleic acid
21	5.47	Elaidic acid
22	15.77	Stearic acid
	100.00	

Figure S3. GC-MS total ion chromatogram (TIC) – Fatty acids composition of amasi.

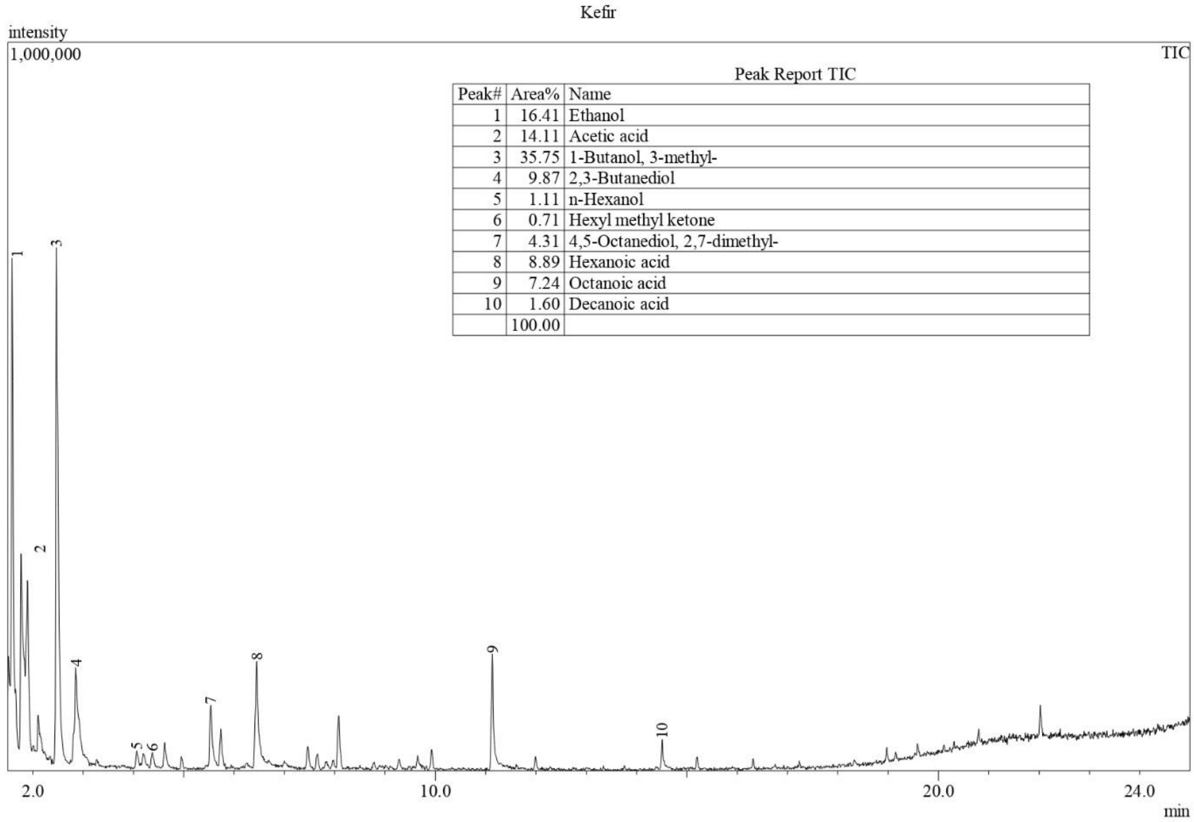

Peak#	Area%	Name
1	16.41	Ethanol
2	14.11	Acetic acid
3	35.75	1-Butanol, 3-methyl-
4	9.87	2,3-Butanediol
5	1.11	n-Hexanol
6	0.71	Hexyl methyl ketone
7	4.31	4,5-Octanediol, 2,7-dimethyl-
8	8.89	Hexanoic acid
9	7.24	Octanoic acid
10	1.60	Decanoic acid
	100.00	

Figure S4. GC-MS total ion chromatogram (TIC) – Volatile organic compounds of kefir.

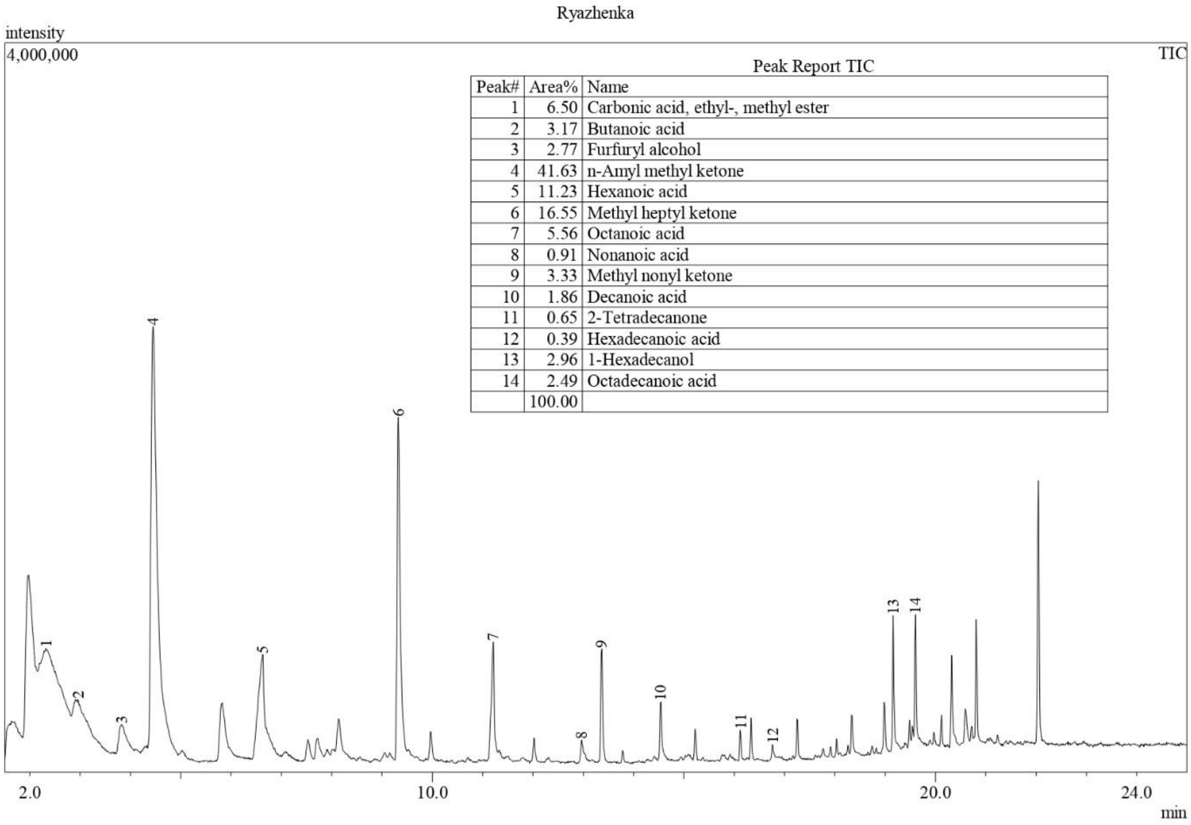

Peak#	Area%	Name
1	6.50	Carbonic acid, ethyl-, methyl ester
2	3.17	Butanoic acid
3	2.77	Furfuryl alcohol
4	41.63	n-Amyl methyl ketone
5	11.23	Hexanoic acid
6	16.55	Methyl heptyl ketone
7	5.56	Octanoic acid
8	0.91	Nonanoic acid
9	3.33	Methyl nonyl ketone
10	1.86	Decanoic acid
11	0.65	2-Tetradecanone
12	0.39	Hexadecanoic acid
13	2.96	1-Hexadecanol
14	2.49	Octadecanoic acid
	100.00	

Figure S5. GC-MS total ion chromatogram (TIC) – Volatile organic compounds of ryazhenka.

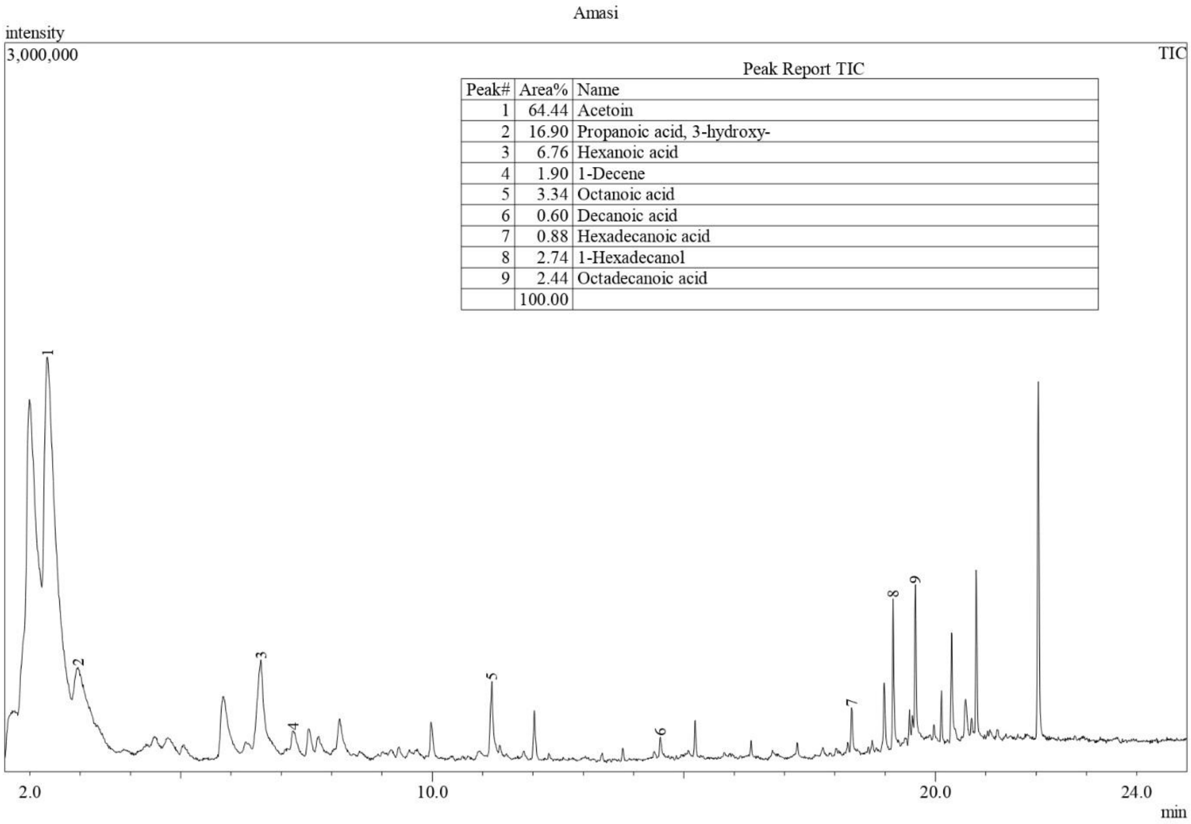

Peak#	Area%	Name
1	64.44	Acetoin
2	16.90	Propanoic acid, 3-hydroxy-
3	6.76	Hexanoic acid
4	1.90	1-Decene
5	3.34	Octanoic acid
6	0.60	Decanoic acid
7	0.88	Hexadecanoic acid
8	2.74	1-Hexadecanol
9	2.44	Octadecanoic acid
	100.00	

Figure S6. GC-MS total ion chromatogram (TIC) – Volatile organic compounds of amasi.

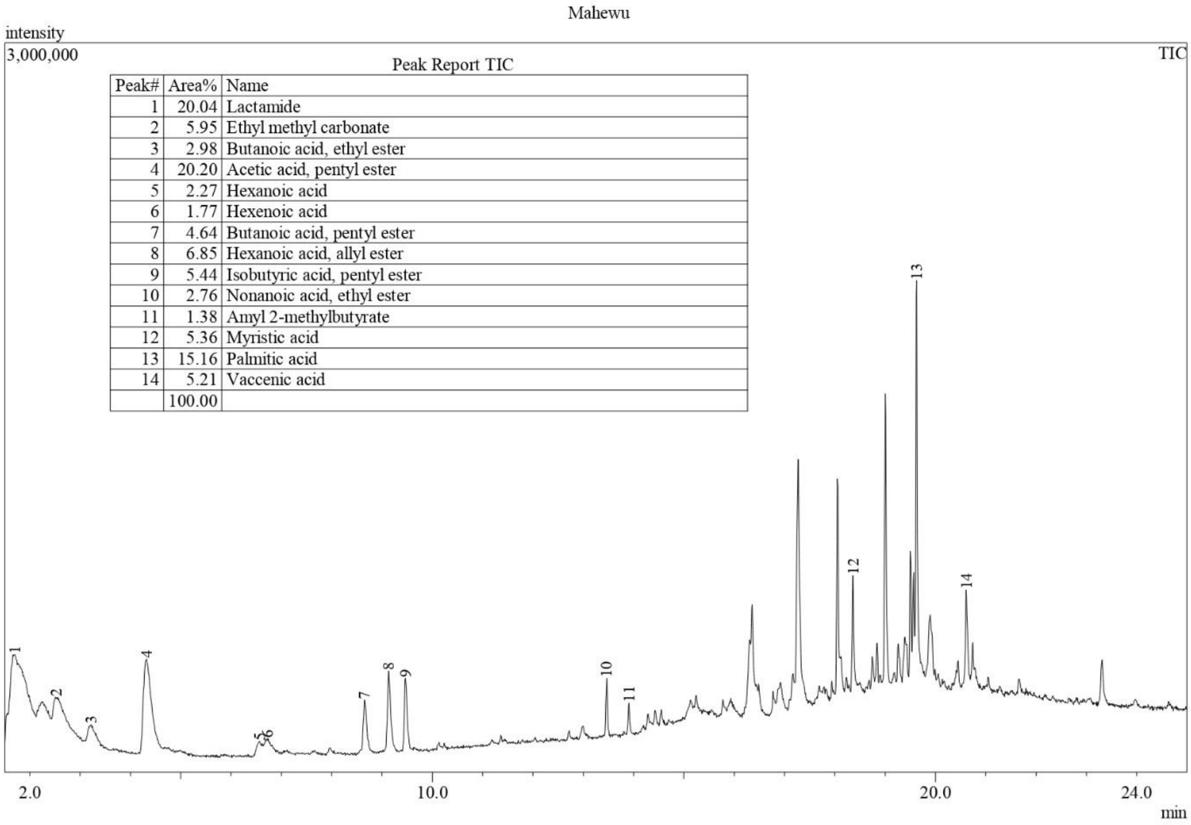

Figure S7. GC-MS total ion chromatogram (TIC) – Volatile organic compounds of mahewu.
